# Effects of Arginine Supplementation on Serum Metabolites and the Rumen Bacterial Community of Sika Deer (*Cervus nippon*)

**DOI:** 10.3389/fvets.2021.630686

**Published:** 2021-02-05

**Authors:** Huazhe Si, Hanlu Liu, Weixiao Nan, Guangyu Li, Zhipeng Li, Yujie Lou

**Affiliations:** ^1^College of Animal Science and Technology, Jilin Agricultural University, Changchun, China; ^2^Department of Special Animal Nutrition and Feed Science, Institute of Special Animal and Plant Sciences, Chinese Academy of Agricultural Sciences, Changchun, China

**Keywords:** sika deer, arginine, IGF-I, *Bacteroides* spp., rumen bacteria

## Abstract

Velvet antler is a regeneration organ of sika deer (*Cervus nippon*) and an important Chinese medicine, and nutrient metabolism affects its growth. Here, we investigated the effects of arginine supplementation on antler growth, serum biochemical indices, and the rumen bacterial community of sika deer during the antler growth period. Fifteen male sika deer (6 years old) were randomly assigned to three dietary groups, which were supplemented with 0 (*n* = 5, CON), 2.5 (*n* = 5, LArg), or 5.0 g/d (*n* = 5, HArg) L-arginine. The IGF-1, ALT and AST concentrations in the serum of LArg sika deer were significantly higher than those in the serum of CON (*P* < 0.05) and HArg deer (*P* < 0.05). The phyla Bacteroidetes, Firmicutes, and Proteobacteria were dominant in the rumen of sika deer among the three groups. Comparison of alpha diversities showed that the ACE and Chao1 indices significantly increased in the LArg and HArg groups compared with those in the CON group. PCoA and ANOSIM results showed that the bacterial community was significantly changed between the CON and LArg groups. Moreover, the relative abundances of *Fibrobacter* spp. and *Prevotellaceae* UCG-003 increased, but those of *Clostridium* sensu stricto 1 and *Corynebacterium* 1 decreased in the LArg and HArg groups compared with those in the CON group. Additionally, the relative abundances of 19 OTUs were significantly different between the LArg and HArg groups. These results revealed that arginine supplementation affected the sika deer rumen bacterial community and serum biochemical indices.

## Introduction

Sika deer (*Cervus nippon*) is an important ruminant species that not only produces traditional Chinese medicine but also provides meat and fur to humans (1). The velvet antler is an organ that can be completely regenerated each year (2, 3). Interestingly, the regeneration rate of velvet antler is even faster than the proliferation of cancer cells. Thus, there are ~800,000 farmed sika deer in China for velvet antler production (4). The improvement of velvet antler production is critical in sika deer productivity.

The growth of velvet antler is affected by several factors, such as genetic factors, dietary conditions and hormones (5, 6). Wang et al. (7) demonstrated that growth factors and receptor genes, including *FGF19, FGF21, FGFBP3, PDGFD*, and *PDGFRL*, were the genetic basis of the rapid growth of velvet antler. In addition, previous studies have demonstrated that insulin-like growth factor-1 (IGF-1) is an important hormone affecting velvet antler growth (8). Arginine is a functional amino acid that plays a key role in urea cycle regulation, hepatic detoxification and protein synthesis. Arginine supplementation increases growth hormone levels in dairy cattle (6) and stimulates the production of luteinizing hormone in prepubertal ewes (9). Interestingly, previous studies showed that supplementation with arginine in the diet increased the IGF-1 concentration in the serum of sheep and cows (10, 11). These results suggested that supplementation with arginine in the diet is likely to increase the concentration of IGF-1 in sika deer and thus improve velvet antler production.

It is well-known that the rumen microbiota plays important roles in the conversion of dietary components, such as proteins and fibers, to volatile fatty acids (VFAs), including acetate, propionate, butyrate, and ammonia, which provide the host with essential nutrients and metabolic energy (12). Of the rumen microbiota constituents, bacteria are the most diverse group and are estimated to be present at 10^9^-10^10^/l (13). Thus, rumen bacteria are a key element for understanding the metabolism of nutrients. Previous studies also demonstrated that supplementation with arginine can increase the molar concentration of acetate and propionate *in vitro* (14). These results suggested that supplementation with arginine in the diet may affect rumen fermentation, which can be attributed to changes in the rumen bacterial community. However, how supplementation with arginine in the diet affects the rumen microbial community of sika deer is still unclear.

Thus, the present study aims to examine the effects of arginine supplementation in the diet (1) on antler production and serum metabolic parameters and (2) on the rumen fermentation and bacterial community of sika deer during the antler growth period.

## Materials and Methods

### Experimental Design, Animals, and Diets

A total of 15 male, 6-year-old sika deer (mean body weight = 94.1 ± 9.70 kg) were used in this study. These sika deer have a similar time point of hard antler button casting around May 15th, 2017. All animal procedures were approved and authorized by the Animal Ethics Committee of Jilin Agricultural University, and Chinese Academy of Agricultural Sciences Animal Care and Use Committee.

A total of 15 sika deer were randomly assigned to three groups, with five animals in each group. Each of the five sika deer in each group was maintained in an individual pen. The sika deer in each group were fed a diet restricted based on corn silage and concentrate (30:70, dry matter basis) and randomly assigned to one of three experimental diets (Table 1): a basal diet with 0 g/d (CON), 2.5 g/d (LArg), or 5.0 g/d (HArg) arginine. All sika deer were fed twice each day at 0700 and 1600 h [a total of 2 kg diet (dry matter)] and had free access to drinking water. The experiments were conducted for 7 weeks after the hard antler button caste, with 1 week for adaptation followed by 6 weeks of dietary treatments.

**Table 1:** Basic dietary composition and nutrient levels (dry matter basis).

**Ingredients**	**(g/100 g)**	**Nutrient levels**	**(%)**
Corn	30.00	DM%	92.57
Soybean meal	20.00	CP%	16.74
Corn fiber	5.00	ME (MJ /kg)	10.78
Distillers dried grains with soluble	5.50	EE%	2.34
Corn germ	8.00	NDF%	58.94
Corn silage	30.00	ADF%	32.54
NaCl	0.50	Arginine	0.98
Premix^1^	1		
Total	100		

*DM, dry matter; CP, crude protein; ME, metabolic energy; EE, ether extract; NDF, neutral detergent fiber; ADF, acid detergent fiber. ME was a calculated value, while the others were measured values. Premix^1^: One kg of premix contained the following: MgO, 0.076 g; ZnSO_4_.H_2_O, 0.036 g; MnSO_4_.H_2_O, 0.043 g; FeSO_4_.H_2_O, 0.053 g; NaSeO_3_, 0.031 g; vitamin A, 2484 IU; vitamin D, 3496.8 IU; vitamin E, 0.828 IU; vitamin K, 0.23 mg; vitamin B_1_, 0.092 mg; vitamin B_2_, 0.69 mg; vitamin B_12_, 0.00138 mg; folic acid, 0.023 mg; nicotinic acid, 1.62 mg; calcium pantothenate, 1.15 mg; CaHPO_4_, 5.17 g; CaCO_3_, 4.57g*.

### Sample Collection

At the end of the experiment (day 50), the animals were euthanized by injection of xylazine hydrochloride (2.0 ml/100 kg body weight) before the morning feeding. After that, velvet antlers were harvested using a sterilized saw under the guidance of the institutional velveting regime, and were weighed (15). Blood samples (10 ml) were collected by puncture of the jugular vein and centrifuged at 4,000 × g for 10 min at 4°C to obtain serum. Rumen liquid (~200 ml) was obtained via the rumen stomach before morning feeding. To avoid saliva contamination, the first 100 ml of rumen fluid was discarded. The samples were transferred into liquid nitrogen and then stored at −80°C for further analysis. The average daily gain of antler was calculated using the following formula: average daily gain (ADG) of antler (g/d) = antler weight/growing time(d).

### Measuring Biochemical Indices in Serum

The concentrations of serum triglycerides (TG), total cholesterol (TC), high-density lipoprotein cholesterol (HDL-C), low-density lipoprotein cholesterol (LDL-C), glucose, total protein(TP), albumin (ALB), globulin (GLB), alkaline phosphatase(ALP), alanine aminotransferase (ALT) and aspartate aminotransferase (AST) were analyzed using commercial colorimetric kits (Nanjing Jiancheng Bioengineering Institute, Jiangsu, China) on a Beckman AU480 automatic biochemistry analyzer (VITALB Selectra E, Netherlands). ELISA kits were used to quantify the concentration of serum IGF-1 (MLbio, Shanghai, China).

### Measuring Amino Acids in Serum

Samples of serum were diluted with trichloroacetic acid and then centrifuged for 5 min at 10,000 × g to precipitate protein, and the supernatant was used directly after centrifugation. The concentrations of serum amino acids were quantified by ion exchange chromatography (Hitachi L8900 amino acid analyzer, Hitachi Technology, Tokyo, Japan) (16).

### Measuring VFAs in Rumen Liquid

The molar concentrations of VFAs in rumen fluid were determined according to a previous study (17). In brief, rumen liquid was centrifuged at 12,000 × g for 10 min at 4°C. Then, 0.2 ml of 2-ethylbutyric acid in meta-phosphoric acid (25% w/v) was added to 1.0 ml of the obtained supernatant. The concentrations of VFAs in the rumen were determined by gas chromatography with a flame ionization detector and a DB-FFAP column (Agilent Technologies 6890GC, CA, USA). The carrier gas was N_2_ at a flow rate of 2.2 ml/min. The analysis was performed with a gradient oven temperature of 80–170°C, an incremental rate of 10°C/min for optimal separation and a detector temperature of 250°C.

### DNA Extraction, Amplification, Sequencing, and Bioinformatics Analysis

The total genomic DNA of microorganisms in rumen liquid was extracted using an MP Fast DNA Spin Kit (MP, Valencia, CA) according to the manufacturer's instructions. The V3–V4 region of the bacterial 16S rRNA gene was amplified using the primers 341F (5′-CCTACGGGAGGCAGCAG-3′) and 806R (5′-GGACTACHVGGGTWTCTAAT-3′). Each primer pair contained the appropriate Illumina adapter sequence and an 8-bp barcode. The resultant amplicons were purified using a QIAquick PCR Purification Kit (QIAGEN, Valencia, CA), which were then sequenced on an Illumina MiSeq platform generating paired 250-bp reads.

The paired sequences were first assembled into contigs using FLASH v1.2.7 (18). The obtained contigs were then processed using quantitative insights into microbial ecology (QIIME v1.9.1) (19). After that, the sequences were clustered into operational taxonomic units (OTUs) using UPARSE at 97% sequence similarity. The possible chimeric sequences were removed using UCHIME (20). The representative sequences of each OTU were assigned against the SILVA database (v138) using the RDP classifier with a 0.80 confidence threshold (21). Alpha-diversity analysis including Chao 1 and ACE indices (richness estimates) and Shannon index (diversity indices) was calculated using QIIME v1.9.1 (19). Principal coordinate analysis (PCoA) was used to analyze the bacterial communities among the three groups (22). Analysis of similarities (ANOSIM) was performed to indicate group similarity, where 0 = indistinguishable and 1 = dissimilar (23). Adonis was employed to describe the strengths and significance of the differences among the microbial communities. For ANOSIM and Adonis analysis, the *P*-values were determined based on 999 permutations. The sequences from the present study have been deposited in the SRA database under accession numbers SRR12210973-SRR12210987.

### Statistical Analysis

One-way analysis of variance (ANOVA) was used to test the statistical significance of alpha diversity indices, antler growth performance, serum biochemical indices, serum amino acids and the relative abundance of bacteria among the three groups. If ANOVA tests indicated a significant difference between means, then Fisher's exact-test was used to determine which of the means differed from each other. All *P*-values were corrected for a false discovery rate (FDR) of 0.05 using the Benjamini–Hochberg method (24), and FDR-corrected *P*-values below 0.05 were considered significant.

## Results

### Antler Weight and Concentrations of Metabolites and Hormones in Serum Among the Three Groups

The results showed that there were no significant differences in the average daily gain and final weight of velvet antler among the three groups (Table 2). As reported in Table 2, the concentration of IGF-1 in serum from the LArg and HArg groups was significantly higher than that in serum from the CON group, but the concentration of AST was lower (*P* < 0.05). Moreover, the concentration of ALT in the LArg group was significantly lower than that in the CON group (*P* < 0.05).

**Table 2:** Effects of arginine supplementation on antler growth performance and serum parameters of sika deer during the antler growth period.

**Item**	**CON**	**LArg**	**HArg**	***P*-value**
Antler weight (g)	1,449 ± 86.2	1,523 ± 91.5	1,436 ± 71.6	0.25
Avarage daily gain of antler (g/d)	28.2 ± 2.11	30.8 ± 1.57	29.2 ± 1.85	0.13
IGF-1 (ng/ml)	101^a^ ± 13.2	190^b^ ± 13.4	179^b^ ± 21.1	<0.01
TG (mmol/l)	0.03 ± 0.02	0.03 ± 0.02	0.04 ± 0.03	0.40
TC (mmol/l)	1.35 ± 0.13	1.44 ± 0.45	1.42 ± 0.24	0.90
HDL-C (mmol/l)	1.23 ± 0.12	1.29 ± 0.42	1.25 ± 0.19	0.95
LDL-C (mmol/l)	0.32 ± 0.06	0.35 ± 0.16	0.36 ± 0.09	0.82
Glucose (mmol/l)	11.0 ± 2.1	7.58 ± 1.97	10.8 ± 3.07	0.08
TP (g/l)	59.9 ± 5.38	58.1 ± 4.55	58.6 ± 3.99	0.74
ALB (g/l)	24.9 ± 1.76	24.3 ± 2.24	24.8 ± 1.35	0.89
GLB (g/l)	35.0 ± 4.01	33.3 ± 3.52	33.3 ± 3.65	0.73
ALP (U/l)	172 ± 91.4	232 ± 119	178 ± 75.1	0.28
ALT (U/l)	62.9^b^ ± 12.9	42.8^a^ ± 12.4	51.5^ab^ ± 12.8	0.08
AST (U/l)	81.0^b^ ± 9.43	58.8^a^ ± 13.83	57.1^a^ ± 7.38	<0.01

*Means with different lowercase of superscripts were significantly different at P < 0.05. IGF-1, Insulin-like growth factor-1; TG, Triglycerides; TC, Total cholesterol; HDL-C, High-density lipoprotein cholesterol; LDL-C, Low-density lipoprotein cholesterol; TP, Total protein; ALB, Albumin; GLB, Globulin; ALP, Alkaline phosphatase; ALT, Alanine aminotransferase; AST, Aspartate aminotransferase*.

### Concentrations of Amino Acids in Serum Among the Three Groups

The results showed that the concentrations of citrulline, sarcosine and ornithine in the HArg group (*P* < 0.05) were significantly higher than those in the CON group, whereas the concentration of ammonia was lower (*P* < 0.05). Moreover, arginine supplementation significantly decreased the concentration of lysine in serum (*P* < 0.05; Table 3). With arginine supplementation, the concentrations of urea and arginine also tended to increase in serum.

**Table 3:** Comparing the concentration of amnio acids of sika deer in serum among the three groups.

**Item(nmol/ml)**	**CON**	**LArg**	**HArg**	***P*-value**
Phosphoserine	1.22 ± 0.44	1.18 ± 0.12	1.23 ± 0.18	0.96
Taurine	2.49 ± 0.95	2.54 ± 0.35	2.04 ± 0.58	0.47
Phosphate ethanolamine	0.84 ± 0.25	0.53 ± 0.40	0.61 ± 0.17	0.31
Urea	159 ± 27.3	167 ± 17.1	182 ± 37.9	0.47
Aspartic acid	0.38 ± 0.10	0.29 ± 0.08	0.32 ± 0.09	0.34
Threonine	0.94 ± 0.12	0.85 ± 0.27	0.97 ± 0.31	0.75
Serine	1.95 ± 0.44	1.84 ± 0.42	2.33 ± 0.50	0.25
Glutamate	2.18 ± 0.61	1.79 ± 0.38	1.79 ± 0.25	0.31
Sarcosine	9.56^b^ ± 5.25	12.85^ab^ ± 2.81	14.99^a^ ± 2.32	0.11
Alpha-aminoadipate	0.14 ± 0.07	0.139 ± 0.02	0.164 ± 0.01	0.46
Glycine	9.61 ± 3.34	10.79 ± 4.03	11.75 ± 3.11	0.64
Alanine	5.01 ± 0.96	6.63 ± 2.36	6.21 ± 0.80	0.27
Citrulline	1.83^b^ ± 0.28	1.91^b^ ± 0.406	3.46^a^ ± 1.66	0.04
α-Aminobutyric acid	0.26 ± 0.15	0.147 ± 0.14	0.17 ± 0.07	0.32
Valine	5.22 ± 1.69	4.35 ± 1.01	5.07 ± 1.19	0.56
Cystine	0.50 ± 0.13	0.44 ± 0.17	0.66 ± 0.03	0.27
Methionine	0.92 ± 0.07	0.94 ± 0.12	0.91 ± 0.16	0.90
Cystathionine	0.26 ± 0.02	0.23 ± 0.07	0.23 ± 0.02	0.57
Isoleucine	1.76 ± 0.38	1.66 ± 0.33	1.69 ± 0.36	0.91
Leucine	2.82 ± 0.68	2.65 ± 0.06	2.90 ± 0.58	0.82
Tyrosine	1.14 ± 0.11	1.21 ± 0.35	1.13 ± 0.16	0.83
Phenylalanine	1.22 ± 0.20	1.11 ± 0.24	1.15 ± 0.24	0.71
Beta alanine	0.86 ± 0.69	0.76 ± 0.70	0.68 ± 0.23	0.89
γ-Aminobutyric acid	0.38 ± 0.27	0.31 ± 0.34	0.24 ± 0.10	0.70
Ethanolamine	1.45 ± 0.61	1.52 ± 0.69	1.29 ± 0.17	0.79
Ammonia	4.54^a^ ± 0.61	4.45^a^ ± 0.48	3.68^b^ ± 0.31	0.03
Ornithine	1.95^b^ ± 0.48	2.16^ab^ ± 0.48	2.56^a^ ± 0.14	0.09
Lysine	2.24^a^ ± 0.47	1.59^b^ ± 0.29	1.76^b^ ± 0.28	0.04
1-methylhistidine	0.42 ± 0.30	0.29 ± 0.25	0.50 ± 0.23	0.48
Histidine	1.26 ± 0.09	1.14 ± 0.18	1.27 ± 0.13	0.27
3-methylhistidine	1.76 ± 0.59	1.18 ± 0.67	2.04 ± 0.98	0.24
Carnosine	0.33 ± 0.12	0.36 ± 0.11	0.36 ± 0.41	0.83
Arginine	2.99 ± 0.56	3.24 ± 0.58	3.82 ± 0.80	0.16
Hydroxyproline	0.45 ± 0.16	0.49 ± 0.21	0.52 ± 0.20	0.80
Proline	1.57 ± 0.12	1.76 ± 0.58	1.74 ± 0.22	0.68

*The values were expressed as mean ± standard deviation (SD). Means with different lowercase of superscripts were significantly different at P < 0.05*.

### Concentrations of Rumen Fermentation Indicators Among the Three Groups

There were no significant differences in molar concentrations of acetate, propionate, butyrate, isobutyrate, isovalerate, valerate, TVFAs and ammonia in the rumen fluid among the three groups (Table 4).

**Table 4:** Effects of arginine supplementation on rumen fermentation parameters of sika deer.

**Item**	**CON**	**LArg**	**HArg**	***P*-value**
Acetate, mmol/l	53.1 ± 10.6	54.1 ± 7.20	51.3 ± 4.69	0.91
Propionate, mmol/l	13.4 ± 3.42	15.3 ± 2.25	14.6 ± 1.77	0.69
Butyrate, mmol/l	8.89 ± 0.91	9.15 ± 0.55	8.41 ± 1.41	0.90
Isobutyrate, mmol/l	1.60 ± 0.34	1.71 ± 0.27	1.73 ± 0.51	0.67
Isovalerate, mmol/l	2.18 ± 0.30	2.47 ± 0.53	2.54 ± 0.59	0.65
Valerate, mmol/l	0.88 ± 0.11	1.11 ± 0.23	1.11 ± 0.30	0.42
TVFAs, mmol/l	80.1 ± 14.6	83.8 ± 9.79	79.7 ± 8.81	0.89
Ammonia, mg/dl	10.6 ± 4.45	11.2 ± 3.74	12.9 ± 2.1	0.59

*The values were expressed as mean ± standard deviation (SD). TVFA, Total volatile fatty acids*.

### Bacterial Community Composition Among the Three Groups

The present study obtained a total of 1,183,284 16S rRNA gene sequences from the three groups, with a range of 50,444 to 93,256 sequences for each sample. We subsampled the sequences in each sample to 50,000 to decrease the effect of sequencing depth. A total of 3,890 OTUs were identified at 97% sequence similarity. Good's coverage ranged from 0.989 to 0.992.

**Figure 1:**
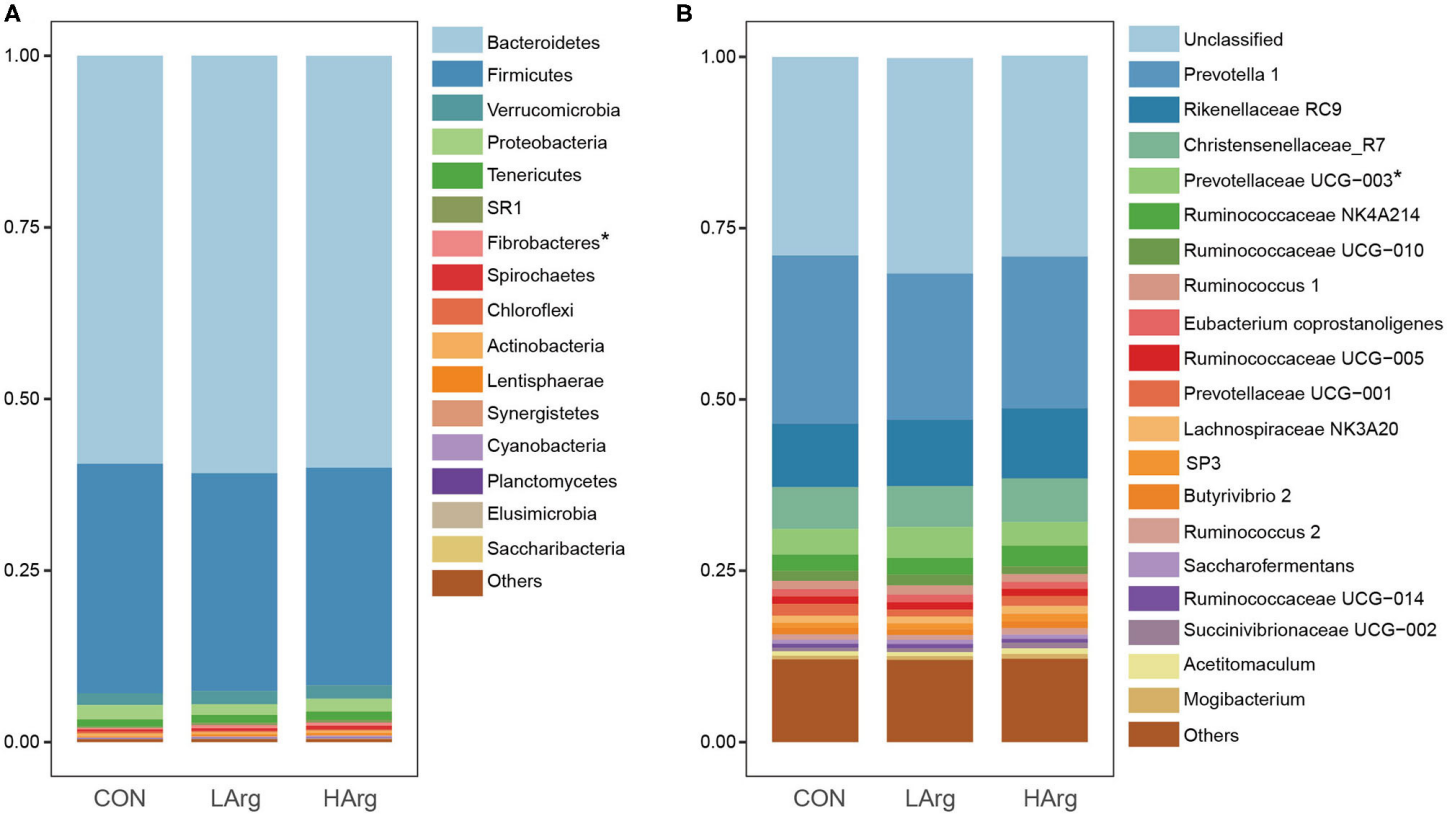
The bacterial composition in the rumen of sika deer at the phylum **(A)** and genus **(B)** levels. *indicate *P* < 0.05.

**Figure 2:**
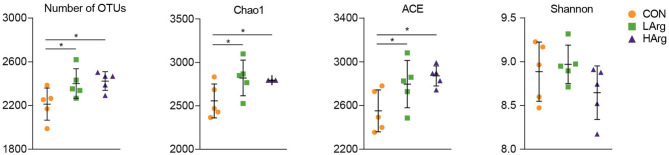
Comparisons of the Alpha diversity of the bacteria in the rumen of sika deer. *indicate *P* < 0.05.

At the phylum level, a total of 25 phyla were identified in the three groups. The phyla Bacteroidetes (CON = 59.3 ± 6.1%, LArg = 60.6 ± 4.9%, and HArg = 59.9 ± 9.8%), Firmicutes (CON = 33.7 ± 5.9%, LArg = 31.7 ± 3.6%, and HArg = 31.7 ± 7.5%), and Proteobacteria (CON = 2.1 ± 0.5%, LArg = 1.6 ± 0.7%, and HArg = 1.8 ± 0.6%) were the most abundant bacteria in rumen fluid among the three groups. At the genus level, *Prevotella* 1, accounting for a more than 20% proportion (CON = 21.8 ± 12.2%, LArg = 24.1.6 ± 5.5%, and HArg = 20.96 ± 9.4%), was the most abundant bacteria in rumen fluid among the three groups. *Rikenellaceae* RC9 (CON = 10.1 ± 2.9%, LArg = 9.3 ± 2.8%, and HArg = 9.6 ± 1.8%), *Christensenellaceae* R7 (CON = 6.1 ± 0.2%, LArg = 5.9 ± 0.1%, and HArg = 5.8 ± 0.2%), *Prevotellaceae* UCG-003 (CON = 3.3 ± 0.8%, LArg = 3.7 ± 0.8%, and HArg = 4.5 ± 0.3%) and *Ruminococcaceae* NK4A214 (CON = 2.9 ± 0.9%, LArg = 2.4 ± 0.3%, and HArg = 2.4 ± 0.7%) were also prevalent in rumen fluid among the three groups (Figure 1). Moreover, in comparison with those in the CON group, the OTU numbers and ACE and Chao1 indices were significantly increased in the LArg and HArg groups (*P* < 0.05, Figure 1); however, the difference between the LArg and HArg groups was not significant (Figure 2).

**Figure 3:**
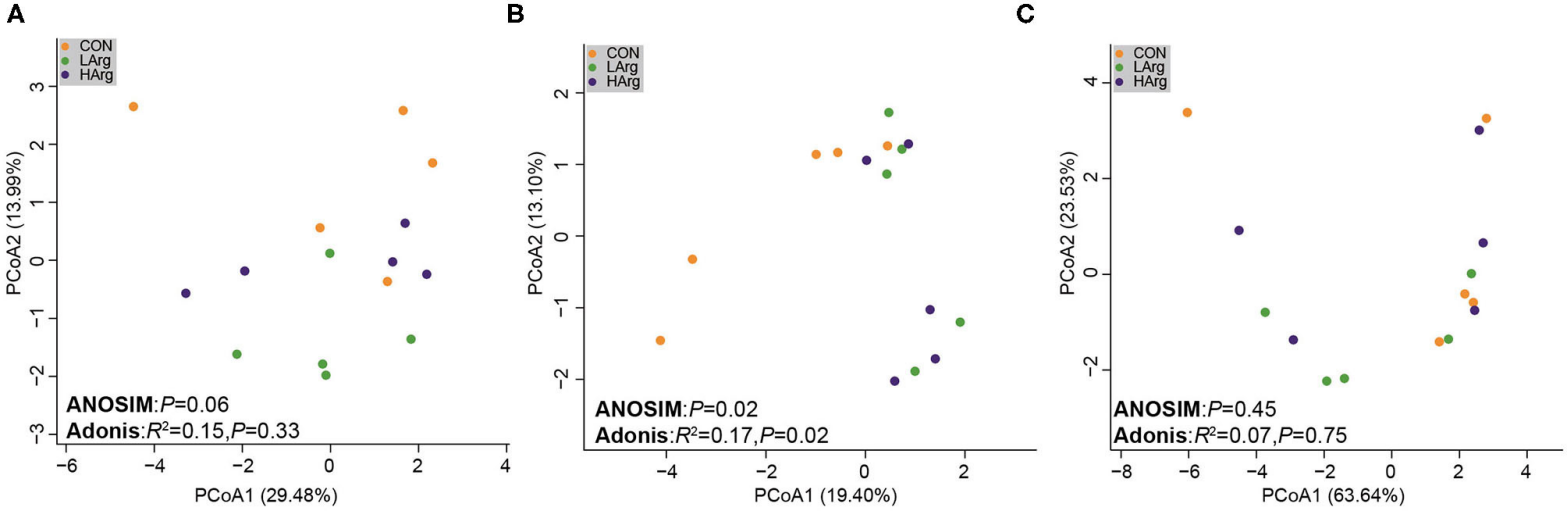
Comparisons of the bacterial communities in the rumen of sika deer. Principal coordinate analyses based on unweighted UniFrac distances **(A)**, Bray cuits distance **(B)** and weighted UniFrac distance **(C)**.

**Figure 4:**
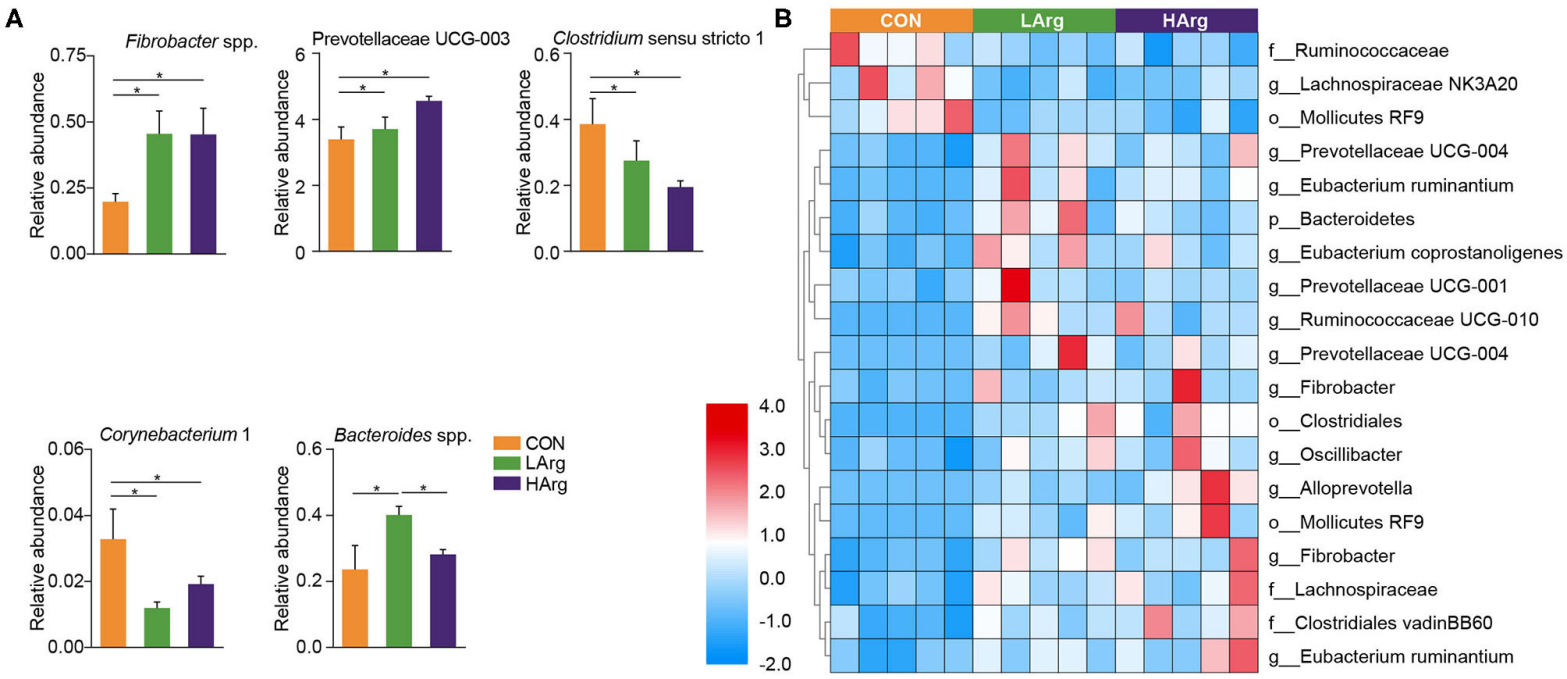
Features characterizing the bacteria in the rumen of sika deer. Bar plots showing differences in the relative abundance of bacteria at genus level across three groups **(A)** and heat map showing the significant differences OTUs in the rumen of LArg and HArg groups comparing to CON group **(B)**. *indicate *P* < 0.05.

The PCoA results showed that the bacterial community composition in the rumen fluid of the LArg and HArg groups was clearly separated from that in the rumen fluid of the CON group based on unweighted UniFrac distance (ANOSIM: *P* = 0.02; Adonis: *R*^2^ = 0.17, *P* = 0.02 Figure 3B). However, the PCoA results based on the Bray-Curtis dissimilarity matrix (ANOSIM: *P* = 0.06; Adonis: *R*^2^ = 0.15, *P* = 0.33 Figure 3A) and weighted UniFrac distance (ANOSIM: *P* = 0.45; Adonis: *R*^2^ = 0.07, *P* = 0.75 Figure 3C) showed no significant differences among the three groups. Furthermore, we also compared the relative abundance of the bacterial genera in rumen fluid among the three groups (Figure 4A). The relative abundances of *Fibrobacter* spp. and *Prevotellaceae* UCG-003 were significantly higher in the LArg and HArg groups than in the CON group. In contrast, the proportions of *Corynebacterium* 1 and *Clostridium* sensu stricto 1 were significantly lower in the LArg and HArg groups than in the CON group. Moreover, the relative abundance of *Bacteroides* spp. was significantly higher in the LArg group than in the CON (0.23 ± 0.16%) and HArg (0.28 ± 0.03%) groups. In contrast with those in the CON group, the relative abundances of 19 OTUs were significantly different between the LArg and HArg groups (Figure 4B). For instance, the proportions of f_Ruminococccaceae, g_Lachnospiraceae NK3A20 and o_Mollicutes RF9 were lower in the LArg and HArg groups than in the CON group; in contrast, the relative abundances of the other 16 OTUs were higher in the LArg and HArg groups than in the CON group.

## Discussion

In this study, the results showed that the concentration of IGF-1 in serum increased with arginine supplementation, with the highest amount in the LArg group (Table 2). Previous studies have shown that arginine stimulates the hypothalamus to release growth hormone (GH) (25), which is one of the most important factors stimulating IGF-I production (26). Yoji Tsugawa et al. (27) demonstrated that arginine can directly increase the IGF-1 concentration in the endoplasmic reticulum of mouse hepatocytes. Together, these results suggested that arginine supplementation improves the production of IGF-1 during the velvet antler growth period of sika deer. In addition, previous studies demonstrated that the concentration of IGF-1 in serum was strongly associated with the growth rate of velvet antler (5). However, the weight and daily gain of velvet antler did not significantly increase as the concentration of IGF-1 increased (Table 2). A previous study also found that it is not IGF-1 but testosterone which is responsible for the intensity of antler growth in subadult and adult red deer (28). Moreover, Romero et al. (29) demonstrated that IGF-1 acts as a negative feedback regulator of growth hormone gene expression when IGF-1 levels reach a certain threshold. These results suggested a possible threshold or signal of IGF-1 concentration in affecting velvet antler growth. Moreover, the limitation of the present study is the small sample size of the animal number, which affected the significant difference among the three groups. Therefore, the effect of changed concentrations of IGF-1 on the growth of sika deer velvet antler needs to be further explored using transcriptome analysis of liver and velvet antler based on a large number of animals, which will provide more direct evidence of the role of arginine in affecting the production of IGF-1 and the growth of velvet antler in future studies.

In this study, the levels of ALT and AST in the serum decreased with arginine supplementation in the diet. Consistent with this finding, Jaja et al. (30) also found that supplementation with arginine in the diet improved liver function and reduced the ALT and AST concentrations in the liver. Interestingly, the liver is also the main site for IGF-1 secretion, and its function directly affects the production of IGF-1. Aksu et al. (31) found that an increased concentration of IGF-1 was strongly associated with decreased concentrations of ALT and AST in serum. Taken together, these results indicated that arginine supplementation in the diet decreased the ALT and AST concentrations in serum.

A previous study revealed that arginine can enter the small intestine and then be converted to citrulline (32). In the liver and kidneys, citrulline is converted to arginine again and then released to surrounding tissues and blood (33). Consistent with these findings, we also found that the concentrations of arginine and citrulline in serum were increased with arginine supplementation in the diet. Moreover, an increase in arginine in serum will promote the urea cycle to hydrolyze arginine and ammonia into urea and ornithine (34), which leads to a decrease in the ammonia concentration and an increase in the urea and ornithine concentrations in blood. Similarly, we found that dietary arginine supplementation caused a decreased concentration of ammonia in blood. In this study, the concentration of lysine in serum decreased in the arginine supplementation groups compared with the CON group (Table 3). Similarly, Teixeira et al. (35) also found a decrease in lysine concentration in the serum when more arginine supplementation was added to diet. Arginine and lysine share common chemical properties and are absorbed in the small intestine by the same transporters; thus, greater supplies of arginine in the small intestine could potentially compete with lysine for transport and cause a decreased supply in the serum (36). Lysine is the first limiting amino acid in most grain- and cereal-based animal diets, and previous studies have also demonstrated the potential for metabolic antagonism between lysine and arginine (37). This is also another possible reason for the non-significant increase in velvet antler production in the arginine supplementation groups. The effects of arginine and lysine on velvet antler growth and production warrant further examination.

The results showed that the genus *Prevotella* 1 was the predominant bacteria across all three groups, which is consistent with previous studies of sika deer (4, 12, 38, 39). *Prevotella* spp. were also widely present in the gastrointestinal tract of other ruminant species, such as roe deer, moose, elk, white lipped deer and reindeer (40–43), and they are involved in the degradation of protein and carbohydrates. In addition, we also found that *Rikenellaceae* RC9, *Christensenellaceae* R7, and *Ruminococcaceae* NK4A214 were prevalent in the rumen fluid of sika deer in this study. Pitta et al. (44) demonstrated that *Rikenellaceae* RC9 was involved in structural carbohydrate degradation. As demonstrated by a metagenomic approach, members of the *Ruminococcaceae* family are important for the degradation of plant fibers (45). *Christensenellaceae* have been shown to play an important role in biofilm formation and rumen degradation of starch and fiber (46). These results suggested the important role of these bacteria in the carbohydrate metabolism of sika deer.

This study also found that the ACE and Chao1 indices were significantly increased by arginine supplementation. The ANOSIM and Adonis analysis based on unweighted UniFrac distance showed that the rumen bacterial community in the rumen fluid of sika deer in the LArg group significantly differed from that in the rumen fluid of sika deer in the CON group. These results suggested that the bacterial community was changed in the rumen fluid of the LArg group. Further comparison of bacterial genera revealed that supplementation with arginine led to a significant increase in the relative abundances of *Fibrobacter* spp. and *Prevotellaceae* UCG-003 and a decrease in the relative abundances of *Clostridium* sensu stricto 1 and *Corynebacterium* 1. Consistent with our observation, Chanthakhoun et al. (47) found that an increased level of dietary protein resulted in an increase in the abundance of *Fibrobacter* spp. Cherdthong et al. (48) reported the relative abundance of *Fibrobacter* Spp. increased with urea supplementation in the diet. Dietary protein is degraded into ammonia in the rumen, which is one of the main sources of nitrogen for *Fibrobacter* spp. Moreover, the concentration of ammonia was slightly increased in the rumen of sika deer from the LArg and HArg groups. Thus, these results indicated that supplementation with arginine in the diet increased the abundance of *Fibrobacter* spp. Consistent with this study, Zhang et al. (49) indicated that dietary L-arginine supplementation inhibited intestinal *Clostridium* spp. colonization in broilers. These results suggested that an arginine supplementation diet is likely to affect the growth of *Clostridium* sensu stricto 1. This study demonstrated that the LArg group had the highest abundance of *Bacteroides* spp. A previous study showed that endogenous arginine flux was positively correlated with the relative abundance of *Bacteroides* spp. (50). This finding may also explain the increased abundance of *Bacteroides* spp. in this study. However, the abundance of *Bacteroides* spp. was not significantly different between the HArg and CON groups. This result may be related to the degradation rate of amino acids in the rumen. Velle et al. (51) indicated that the degradation rate of high-dose amino acids in the rumen was greatly reduced compared with that after low-dose amino acid supplementation.

Moreover, we further examined the significantly differentially abundant OTUs among the three groups. For instance, the abundances of g__Prevotellaceae UCG-004, g__Prevotellaceae UCG-001, o__Clostridiales, g__Prevotellaceae UCG-004, g__Fibrobacter and g__Alloprevotella were increased in the LArg and HArg groups, which were close to *Eubacterium xylanophilum* (90.91%, similarity), *Treponema bryantii* (90.51%, similarity), *Ruminococcus flavefaciens* (83.34%, similarity), *Prevotella* (94.90%, similarity), *Prevotella* (95.27%, similarity) and *Saccharofermentans acetigenes* (95.24%, similarity). *Eubacterium xylanophilum* ferments only cellobiose, xylan and aesculin (52). *Treponema bryantii* uses soluble sugars released from cellulose by cellulolytic bacteria (53). *Ruminococcus flavefaciens* is a predominant cellulolytic rumen bacterium that forms a multienzyme cellulose complex that plays an integral role in the ability of this bacterium to degrade plant cell wall polysaccharides (54). *Saccharofermentans acetigenes* ferments several hexoses, polysaccharides, and alcohols (55). Together, these findings further suggested that arginine supplementation in the diet may affect the degradation of structural or non-structural carbohydrates in the rumen of sika deer. However, this observation needs to be further examined using the shotgun metagenomics approach. The present study found no significant differences in concentrations of VFAs among three groups (Table 4). Moreover, the molar concentrations of VFAs in our study were lower than the previous findings (12, 17, 56), which is likely related to the time point of collecting rumen liquid, and the rumen physiology of sika deer (57).

## Conclusion

In the present study, we investigated the effect of arginine supplementation in the diet on the antler growth performance of sika deer and found that supplementation with arginine affected the production of IGF-1. Moreover, the rumen bacterial composition of sika deer also changed with supplementation with arginine in the diet.

## Data Availability Statement

The datasets generated in this study can be found in online repositories. The names of the repository/repositories and accession number(s) can be found below: https://www.ncbi.nlm.nih.gov/, SRR12210973-SRR12210987.

## Ethics Statement

All animal procedures were approved and authorized by the Animal Ethics Committee of Jilin Agricultural University, and Chinese Academy of Agricultural Sciences Animal Care and Use Committee.

## Author Contributions

ZL and YL: conceptualization. HL: methodology. HS: software and visualization. HS and WN: data curation and writing original draft preparation. ZL: writing review and editing. YL: supervision. ZL and HL funding acquisition. All authors: have read and agreed to the published version of the manuscript.

## Conflict of Interest

The authors declare that the research was conducted in the absence of any commercial or financial relationships that could be construed as a potential conflict of interest.

## References

[1] Hayashida M Souma K Sugo K Araki S Ishizaka F Ueda M. Sex and age differences in meat composition of Yeso sika deer (*Cervus nippon yesoensis*) reared for a short period after capture in the wild. Anim Sci J. (2015) 86:207–13. 10.1111/asj.12274 25186458

[2] Li CY Zhao HP Liu Z McMahon C. Deer antler—A novel model for studying organ regeneration in mammals. Int J Biochem Cell B. (2014) 56:111–22. 10.1016/j.biocel.2014.07.007 25046387

[3] Li CY. Deer antler regeneration: a stem cell-based epimorphic process. Birth Defects Res C. (2012) 96:51–62. 10.1002/bdrc.21000 22457177

[4] Li Z Wright AG Liu H Fan Z Yang F Zhang Z. Response of the rumen microbiota of sika deer (*Cervus nippon*) fed different concentrations of tannin rich plants. PLoS ONE. (2015)10:e0123481. 10.1371/journal.pone.0123481 25955033 PMC4425498

[5] Fennessy P Suttie J Antler growth: nutritional and endocrine factors. Biology of deer production. NZ Royal Soc. (1985) 1:239–50.

[6] Anderson SJ Côté SD Richard JH Shafer AB Genomic architecture of artificially and sexually selected traits in a wild cervid. bioRxiv. (2019) 12:841528 10.1101/841528

[7] Wang Y Zhang C Wang N Li Z Heller R Liu R. Genetic basis of ruminant headgear and rapid antler regeneration. Science. (2019) 364:6446. 10.1126/science.aav6335 31221830

[8] Sui Z Yuan H Liang Z Zhao Q Wu Q Xia S. An activity-maintaining sequential protein extraction method for bioactive assay and proteome analysis of velvet antlers. Talanta. (2013) 107:189–94. 10.1016/j.talanta.2013.01.015 23598211

[9] Recabarren SE Jofré A Lobos A Orellana P Parilo J. Effect of arginine and ornithine infusions on luteinizing hormone secretion in prepubertal ewes. J Anim Sci. (1996) 74:162–6. 10.2527/1996.741162x 8778095

[10] Sano H Nakamura S Kobayashi S Takahashi H Terashima Y. Effect of cold exposure on profiles of metabolic and endocrine responses and on responses to feeding and arginine injection in sheep. J Anim Sci. (1995) 73:2054–62. 10.2527/1995.7372054x 7592091

[11] Chew BP Eisenman JR Tanaka TS. Arginine infusion stimulates prolactin, growth hormone, insulin, and subsequent lactation in pregnant dairy cows. J Dairy Sci. (1984) 67:2507–18. 10.3168/jds.S0022-0302(84)81607-0 6394628

[12] Li Z Wright ADG Liu H Bao K Zhang T Wang K. Bacterial community composition and fermentation patterns in the rumen of sika deer (*Cervus nippon*) fed three different diets. Microb Ecol. (2015) 69:307–18. 10.1007/s00248-014-0497-z 25252928

[13] Henderson G Cox F Ganesh S Jonker A Young W Janssen PH Rumen microbial community composition varies with diet and host, but a core microbiome is found across a wide geographical range. Sci Rep. (2015) 5:14567 10.1038/srep14567 26449758 PMC4598811

[14] Chacher B Wang DM Liu HY Liu JX Degradation of L-arginine and N-carbamoyl glutamate and their effect on rumen fermentation *in vitro*. Ital J Anim Sci. (2012) 11:e68 10.4081/ijas.2012.e68

[15] Sun W Zhao H Bao K Li CY Li GY Dietary calcium supplementation affects nutrient digestibility and antler-production performance during the antler-velvet growth period of male sika deer. Anim Prod Sci. (2019) 59:1689–95. 10.1071/AN17862

[16] Huang J Zhang TT Bao K Li GY Wang KY Effect of supplementation of lysine and methionine on growth performance, nutrients digestibility and serum biochemical indices for growing sika deer (*Cervus nippon*) fed protein deficient diet. Ital J Anim Sci. (2015) 14:60–5. 10.4081/ijas.2015.3640

[17] Li Z André-Denis GW Si H Wang X Qian W Zhang Z. Changes in the rumen microbiome and metabolites reveal the effect of host genetics on hybrid crosses. Environ Microbiol Rep. (2016) 8:1016–23. 10.1111/1758-2229.12482 27717170

[18] Tanja M Salzberg SL. FLASH: fast length adjustment of short reads to improve genome assemblies. Bioinformatics. (2011) 27:2957–63. 10.1093/bioinformatics/btr507 21903629 PMC3198573

[19] Caporaso JG Kuczynski J Stombaugh J Bittinger K Bushman FD Costello EK. QIIME allows analysis of high-throughput community sequencing data. Nat Methods. (2010) 7:335–6. 10.1038/nmeth.f.303 20383131 PMC3156573

[20] Edgar RC. UPARSE: highly accurate OTU sequences from microbial amplicon reads. Nat Methods. (2013) 10:996. 10.1038/nmeth.2604 23955772

[21] Christian Q Elmar P Pelin Y Jan G Timmy S Pablo Y. The SILVA ribosomal RNA gene database project: improved data processing and web-based tools. Nucleic Acids Res. (2013) 41:590–6. 10.1093/nar/gks1219 23193283 PMC3531112

[22] DufreNe M Legendre P Species assemblages and indicator species: the need for a flexible asymmetrical approach. Ecol Monogr. (1997) 67:345–66. 10.2307/2963459

[23] Fierer N Lauber CL Zhou N McDonald D Costello EK Knight R. Forensic identification using skin bacterial communities. Proc Natl Acad Sci. (2010) 107:6477–81. 10.1073/pnas.1000162107 20231444 PMC2852011

[24] Benjamini Y Hochberg Y On the adaptive control of the false discovery rate in multiple testing with independent statistics. J Educ Behav Stat. (2000) 25:60–83. 10.3102/10769986025001060

[25] Alba-Roth J Muller OA Schopohl J von Werder K. Arginine stimulates growth hormone secretion by suppressing endogenous somatostatin secretion. J Clin Endocrinol Metab. (1988) 67:1186–9. 10.1210/jcem-67-6-1186 2903866

[26] Houston B. Insulin and growth hormone act synergistically to stimulate insulin-like growth factor-I production by cultured chicken hepatocytes. J Endocrinol. (1991) 128:389–93. 10.1677/joe.0.1280389 2013745

[27] Tsugawa Y Handa H Imai T. Arginine induces IGF-1 secretion from the endoplasmic reticulum. Biochem Biophys Res Commun. (2019) 514:1128–32. 10.1016/j.bbrc.2019.05.044 31101333

[28] Bartoš L Schams D Bubenik GA. Testosterone, but not IGF-1, LH, prolactin or cortisol, may serve as antler-stimulating hormone in red deer stags (*Cervus elaphus*). Bone. (2009) 44:691–8. 10.1016/j.bone.2008.12.004 19124089

[29] Romero CJ Pine-Twaddell E Sima DI Miller RS He L Wondisford F. Insulin-Like growth factor 1 mediates negative feedback to somatotroph GH expression via POU1F1/CREB binding protein interactions. Mol Cell Biol. (2012) 32:4258–69. 10.1128/mcb.00171-12 22890843 PMC3486141

[30] Jaja SI Ogungbemi SO Kehinde MO Anigbogu CN. Supplementation with L-arginine stabilizes plasma arginine and nitric oxide metabolites, suppresses elevated liver enzymes and peroxidation in sickle cell anaemia. Pathophysiology. (2016) 23:81–5. 10.1016/j.pathophys.2016.04.004 27156372

[31] Aksu I Baykara B Kiray M Gurpinar T Sisman AR Ekerbicer N. Serum IGF-1 levels correlate negatively to liver damage in diabetic rats. Biotech Histochem. (2013) 88:194–201. 10.3109/10520295.2012.758311 23331186

[32] Cynober L Boucher JL Vasson MP Arginine metabolism in mammals. J Nutr Biochem. (1995) 6:402–13. 10.1016/0955-2863(95)00066-9

[33] Wu G Bazer FW Davis TA Kim SW Li P Rhoads JM. Arginine metabolism and nutrition in growth, health and disease. Amino Acids. (2009) 37:153–8. 10.1007/s00726-008-0210-y 19030957 PMC2677116

[34] Barbul A. Arginine: biochemistry, physiology, and therapeutic implications. Jpen J Parenter Enteral Nutr. (1986) 10:227–38. 10.1177/0148607186010002227 3514981

[35] Teixeira PD Tekippe JA Rodrigues LM Ladeira MM Pukrop JR Kim YB Effect of ruminally protected arginine and lysine supplementation on serum amino acids, performance, and carcass traits of feedlot steers. J Anim Sci. (2019) 97:3511–22. 10.1093/jas/skz191 31175366 PMC6667248

[36] Batista ED Hussein AH Detmann E Miesner MD Titgemeyer EC. Efficiency of lysine utilization by growing steers. J Anim Sci. (2016) 42:648–55. 10.2527/jas.2015-9716 27065135

[37] Ball RO Urschel KL Pencharz PB. Nutritional consequences of interspecies differences in arginine and lysine metabolism. J Nutr. (2007)137:1626S−41S. 10.1093/jn/137.6.1626S 17513439

[38] Li Z Zhang Z Chao X Zhao J Liu H Fan Z. Bacteria and methanogens differ along the gastrointestinal tract of chinese roe deer (*Capreolus pygargus*). PLoS ONE. (2014) 9:e114513. 10.1371/journal.pone.0114513 25490208 PMC4260832

[39] Li ZP Henderson G Yang YH Li GY. Diversity of formyltetrahydrofolate synthetase genes in the rumens of roe deer (*Capreolus pygargus*) and sika deer (*Cervus nippon*) fed different diets. Can J Microbiol. (2017) 63:11–9. 10.1139/cjm-2016-0424 27819479

[40] Ishaq SL Wright ADG. Insight into the bacterial gut microbiome of the North American moose (*Alces alces*). BMC Microbiol. (2012) 12:1–12. 10.1186/1471-2180-12-212 22992344 PMC3585231

[41] Lisa MG Diane O Kang AYH Maguire AJ Marco K Klieve AV. Shedding light on the microbial community of the macropod foregut using 454-amplicon pyrosequencing. PLoS ONE. (2013) 8:e61463. 10.1371/journal.pone.0061463 23626688 PMC3634081

[42] Shepherd ML Swecker WS Jr Jensen RV Ponder MA. Characterization of the fecal bacteria communities of forage-fed horses by pyrosequencing of 16S rRNA V4 gene amplicons. FEMS Microbiol Lett. (2012) 326:62–68. 10.1111/j.1574-6968.2011.02434.x 22092776

[43] Zened A Combes S Cauquil L Mariette J Klopp C Bouchez O. Microbial ecology of the rumen evaluated by 454 GS FLX pyrosequencing is affected by starch and oil supplementation of diets. FEMS Microbiol Ecol. (2013) 83:504–14. 10.1111/1574-6941.12011 22974422

[44] Pitta DW Pinchak WE Dowd SE Osterstock J Gontcharova V Youn E. Rumen bacterial diversity dynamics associated with changing from bermudagrass hay to grazed winter wheat diets. Microb Ecol. (2010) 59:511–22. 10.1007/s00248-009-9609-6 20037795

[45] Kim H Lee I Kwon Y Kim BC Su H Lee JH. Immobilization of glucose oxidase into polyaniline nanofiber matrix for biofuel cell applications. Biosens Bioelectron. (2011) 26:3908–13. 10.1016/j.bios.2011.03.008 21470844

[46] Mao SY Huo WJ Zhu WY. Microbiome–metabolome analysis reveals unhealthy alterations in the composition and metabolism of ruminal microbiota with increasing dietary grain in a goat model. Environ Microbiol. (2015) 18:525–41. 10.1111/1462-2920.12724 25471302

[47] Chanthakhoun V Wanapat M Berg J Level of crude protein in concentrate supplements influenced rumen characteristics, microbial protein synthesis and digestibility in swamp buffaloes (*Bubalus bubalis*). Livest Sci. (2012) 144:197–204. 10.1016/j.livsci.2011.11.011

[48] Cherdthong A Wanapat M Wachirapakorn C. Influence of urea-calcium mixtures as rumen slow-release feed on *in vitro* fermentation using a gas production technique. Arch Anim Nutr. (2011) 65:242–54. 10.1080/1745039X.2011.568277 21776840

[49] Zhang B Lv Z Li H Guo S Liu D Guo Y. Dietary L-arginine inhibits intestinal *Clostridium perfringens* colonisation and attenuates intestinal mucosal injury in broiler chickens. Brit J Nutr. (2017) 118:321. 10.1017/S0007114517002094 28901890

[50] Kao CC Cope JL Hsu JW Dwarkanath P Karnes JM Luna RA. The microbiome, intestinal function, and arginine metabolism of healthy indian women are different from those of american and jamaican women. J Nutr. (2015) 146:706–13. 10.3945/jn.115.227579 26962180

[51] Velle W Sjaastad OV Aulie A Gronset D Feigenwinter K Framstad T. Rumen escape and apparent degradation of amino acids after individual intraruminal administration to cows. J Dairy Sci. (1997) 80:3325–32. 10.3168/jds.S0022-0302(97)76308-2 9436115

[52] Toorn JJTK Gylswyk NOV Xylan-digesting bacteria from the rumen of sheep fed maize straw diets. J Gen Microbiol. (1985) 131:2601–07. 10.1099/00221287-131-10-2601

[53] Stanton T Canale-Parola E. *Treponema bryantii* sp. nov., a rumen spirochete that interacts with cellulolytic bacteria. Arch Microbiol. (1980) 127:145–56. 10.1007/BF00428018 7425785

[54] Miller MEB Antonopoulos DA Rincon MT Band M Bari A Akraiko T. Diversity and strain specificity of plant cell wall degrading enzymes revealed by the draft genome of *Ruminococcus flavefaciens* FD-1. PLoS ONE. (2009) 4:e6650. 10.1371/journal.pone.0006650 19680555 PMC2721979

[55] Chen S Niu L Zhang Y. *Saccharofermentans acetigenes* gen. nov., sp. nov., an anaerobic bacterium isolated from sludge treating brewery wastewater. Int J Syst Evol Microbiol. (2010) 60:2735. 10.1099/ijs.0.017590-0 20061495

[56] Patra AK Park T Braun HS Geiger S Pieper R Yu Z. Dietary bioactive lipid compounds rich in menthol alter interactions among members of ruminal microbiota in sheep. Front Microbiol. (2019) 10:2038. 10.3389/fmicb.2019.02038 31551974 PMC6738200

[57] Asano S Ikeda S Kasuya N Kurokawa Y Kanda S Itabashi H Comparative digestion of cell wall components of alfalfa hay cubes between Sika deer (*Cervus nippon*) and cattle. Ani Sci J. (2008) 79:35–40. 10.1111/j.1740-0929.2007.00495.x

